# Onset of Hyperkalemia following the Administration of Angiotensin-Converting Enzyme Inhibitor or Angiotensin II Receptor Blocker

**DOI:** 10.1155/2021/5935149

**Published:** 2021-01-22

**Authors:** Hye-Ran Jun, Hyunah Kim, Seung-Hwan Lee, Jae Hyoung Cho, Hyunyong Lee, Hyeon Woo Yim, Kun-Ho Yoon, Hun-Sung Kim

**Affiliations:** ^1^Department of Psychiatry, College of Medicine, The Catholic University of Korea, Seoul, Republic of Korea; ^2^College of Pharmacy, Sookmyung Women's University, Seoul, Republic of Korea; ^3^Division of Endocrinology and Metabolism, Department of Internal Medicine, Seoul St. Mary's Hospital, College of Medicine, The Catholic University of Korea, Seoul, Republic of Korea; ^4^Department of Medical Informatics, College of Medicine, The Catholic University of Korea, Seoul, Republic of Korea; ^5^Clinical Research Coordinating Center, Catholic Medical Center, The Catholic University of Korea, Republic of Korea; ^6^Department of Preventive Medicine, College of Medicine, The Catholic University of Korea, Republic of Korea

## Abstract

**Introduction:**

In spite of the established importance of detecting angiotensin-converting enzyme inhibitor (ACEI) or angiotensin II receptor blocker- (ARB-) induced hyperkalemia, there have not been many studies on the time of its occurrence.

**Methods:**

We retrospectively analyzed electronic medical records to determine the onset time and incidence rate of hyperkalemia (serum potassium > 5.5 mEq/L or 6.0 mEq/L) among hospitalized patients newly started on a 15-day ACEI or ARB therapy.

**Results:**

Among 3101 hospitalized patients, hyperkalemia incidence was 0.5%–0.9% and 0.8%–2.1% in the ACEI and ARB groups, respectively. However, it was not significantly different among different ARB types. Hyperkalemia's onset was distributed throughout 15 days, without any trend. Hyperkalemia incidence was 7.3 and 35.1 times higher at 5.5 mEq/L (hazard ratio (HR) = 7.31, 95%confidence interval (CI) = 4.19–12.76, *p* < 0.001) and 6.0 mEq/L (HR = 35.11, 95%CI = 8.25–149.52, *p* < 0.001), respectively, than the baseline creatinine level. Hyperkalemia incidence in patients with chronic renal failure was 5.7 and 9.2 times higher at 5.5 mEq/L (HR = 5.72, 95%CI = 3.24–10.12, *p* < 0.001) and 6.0 mEq/L (HR = 9.16, 95%CI = 4.02–20.88, *p* < 0.001), respectively.

**Conclusions:**

It is unlikely that it is necessary to monitor hyperkalemia immediately after administration of ACEI or ARB. However, when prescribed for patients with abnormal kidney function, clinicians should always consider the possibility of developing hyperkalemia.

## 1. Introduction

Angiotensin-converting enzyme inhibitors (ACEIs) and angiotensin II receptor blockers (ARBs) are the most widely used antihypertensive drugs worldwide [1]. They inhibit the renin-angiotensin-aldosterone system (RAAS) and impart HDCDT_5935149pleiotropic effects such as blood pressure control as well as protection against proteinuria, microalbuminuria [2], and cardiovascular disease [3]. Although ACEI or ARB administration has a variety of advantages, side effects such as dry cough, angioedema, and hyperkalemia must be monitored [4].

The serum potassium level is often not monitored after ACEI or ARB administration because the incidence of hyperkalemia after ACEI or ARB initiation is less than 2% and potassium levels are known to rise to within 0.1–0.2 mmol/L [5]. Hence, hyperkalemia is more likely to be found incidentally through other tests [6] or complaints of nonspecific symptoms such as orthostatic hypotension, helplessness, nausea, and fatigue rather than through regular monitoring [7]. However, in some individuals, hyperkalemia can be detrimental enough to require first aid or inpatient treatment, and hence, the risks of hyperkalemia should be adequately recognized [8]. Despite the relatively low incidence, it is recommended that baseline serum potassium levels be checked and glomerular filtration rate (GFR) be estimated before initiating ACEI or ARB, considering the risk of hyperkalemia in patients [9].

Despite the established importance of detecting ACEI- or ARB-induced hyperkalemia, few studies have examined the timing of its occurrence. The timing of hyperkalemia occurrence and its monitoring after ACEI or ARB initiation is an important issue. A previous study reported that more than 50% of hospitalized patients demonstrated ARB-induced hyperkalemia within 1 week of treatment initiation, and with the highest incidence occurring within 24 hours [10]. The authors thus concluded that the monitoring time for early detection of hyperkalemia should not exceed one week, and high-risk patients should be monitored as soon as possible after treatment initiation.

However, the onset time of hyperkalemia after ACEI or ARB administration remains poorly studied. We analyzed the incidence of ARB-induced hyperkalemia among inpatients who received ARB for the first time during their hospitalization. In addition to the onset time of hyperkalemia, we investigated the risk factors that affect its occurrence.

## 2. Methods

### 2.1. Study Design

From January 2009 to December 2015, a total of 7405 patients were admitted to the Catholic University Seoul St. Mary's Hospital who were newly administrated ACEI or ARB and were studied. Records of age, sex, height, weight, blood urea nitrogen (BUN), creatinine, sodium, and potassium at the time of the first administration of ACEI (or ARB) were obtained. The serum potassium level measured on the first day of administration of ACEI (or ARB) treatment during hospitalization was defined as the level on “day 0.” Serum potassium levels for all patients were measured for 15 days from the start date of ACEI (or ARB) administration. In cases where the serum potassium levels were measured multiple times a day, the first reading of the day was selected, and levels that were beyond the normal range of serum potassium levels (<3.5 mEq/L or >5.0 mEq/L) were excluded. Hyperkalemia was divided into two categories, levels over 5.5 mEq/L and 6.0 mEq/L, and the cumulative incidence of hyperkalemia after ACEI or ARB use for 15 days was examined. GFR was calculated using the standard formulation of the modification of diet in renal disease-GFR (MDRD-GFR) value [11].

MDRD − eGFR (mL/min/1.73 m^2^) = 186 × Pcr (mg/dL)^−1.154^ × age^−0.203^ × 0.742 (if female).

The presence of underlying diseases such as heart failure (I50, I11.0, I13.0, I24.8), hypertensive diseases (I10-15), ischemic heart diseases (IHD) (I20-25), cerebrovascular diseases (I60-69), diabetes mellitus (E10-15), acute renal failure (N179), and cancer (C-) at the time of admission was assessed using ICD-10 classification. Further, we investigated the use of statins and other antihypertensive drugs like *β*-blockers, calcium channel blockers (CCB), and diuretics.

### 2.2. Type of ACEIs/ARBs

The ACEIs administrated at Seoul St. Mary's Hospital were as follows: captopril (12.5 mg, 25 mg, or 50 mg), enalapril (5 mg or 10 mg), ramipril (2.5 mg, 5 mg, or 10 mg), lisinopril (10 mg), imidapril (5 mg or 10 mg), and moexipril (7.5 mg or 15 mg). The ARBs administrated at Seoul St. Mary's Hospital were as follows: candesartan (8 mg, 16 mg, 32 mg), valsartan (80 mg, 160 mg), fimasartan (30 mg, 60 mg, 120 mg), irbesartan (150 mg, 300 mg), olmesartan (10 mg, 20 mg, 40 mg), telmisartan (40 mg, 80 mg), and eprosartan (600 mg).

### 2.3. Data Quality Management

Data quality management was performed for statistical analysis after data extraction. The extracted data that contained nonstandardized numeric characters (e.g., *K* = 4.5 (hemolysis)) was excluded, and in such a case, a direct chart review was performed, and patients with hyperkalemia were examined by direct chart review to see if there were other causes.

### 2.4. Protection of Personal Privacy

The patient registration numbers were deleted, and temporary numbers were assigned in the data extracted for the study. This data file was stored in an encrypted form on the encrypted computer of the corresponding author, thus enabling access and viewing of data by the corresponding author only. This data published in this study belongs to patients who have already been discharged and which was collected at the end of the treatment, so this study is not in a violation of the rights and interests of the participants and has little impact on their mental or physical health. The study was approved by the institutional review board of the Catholic University of Korea. Therefore, informed consent was not required.

### 2.5. Statistical Analysis

Descriptive statistics are presented as means and standard deviations or percentages of participants. Kaplan-Meier survival curve analysis and log-rank tests were performed to compare the incidence of hyperkalemia and antihypertensive drug use. The causal relationship between hyperkalemia incidence and baseline variables was analyzed by univariate and multivariate Cox proportional hazard analyses considering multicollinearity. All analyses were performed with the use of the SAS software, version 9.4 (SAS Institute Inc., Cary, NC), and a two-sided *p* < 0.05 was considered statistically significant.

## 3. Results

**Figure 1:**
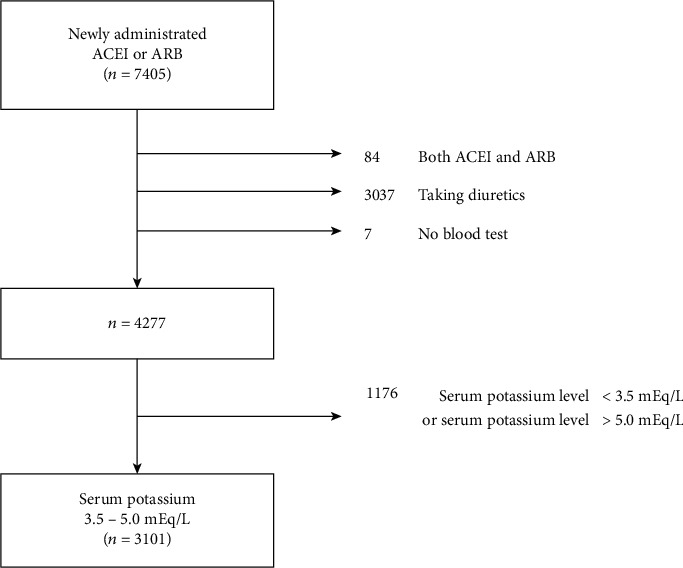
Selection of hospital inpatients who were starting angiotensin-converting enzyme inhibitor or angiotensin receptor blocker use.

The final study population was chosen from the 7405 patients admitted in total. Of the 7405 patients, 84 who took both ACEIs and ARBs were excluded. When 3.5–5.0 mEq/L was considered the normal potassium level, 1176 patients with abnormal potassium levels at baseline before ACEI or ARB administration were excluded. In addition, 3037 patients who were administrated diuretics were also excluded because this study monitors changes in serum potassium levels. Finally, after excluding 7 patients who were administrated ACEI (or ARB) without a blood test, for hyperkalemia at >5.5 mEq/L, 3101 patients were enrolled in the study (Figure 1).

### 3.1. Baseline Characteristics according to ACEI and ARB Use

The baseline characteristics of 3101 hospitalized patients starting treatment with an ACEI or ARB are presented in Table 1. ACEIs were administrated to 18.1% of the patients (561/3101) and ARBs to 81.9% (2539/3101). The average age of patients in the ACEI group was 62 ± 12 years and 63 ± 12 years in the ARB group. The BMI of patients was 23.8 ± 3.3 kg/m^2^ in the ACEI group and 24.5 ± 3.6 kg/m^2^ in the ARB group. MDRD-eGFR was 82.1 ± 24.1 mL/min/1.73 m^2^ in the ACEI group and 76.3 ± 26.9 mL/min/1.73 m^2^ in the ARB group. Percentages of patients who had levels of MDRD-eGFR above 60 mL/min/1.73 m^2^ were 86.5% and 79.4% in the ACEI and ARB groups, respectively. Serum potassium levels showed a statistically significant difference between the two groups (4.2 ± 0.4 vs. 4.3 ± 0.5, *p* = 0.009), but this was not clinically significant. There was a significant difference between the two groups regarding cases of IHD, including angina (53.9% vs. 38.5%, *p* < 0.001), acute renal failure (1.3% vs. 4.3%, *p* = 0.001), and cancer (7.7% vs. 10.8%, *p* < 0.05). There were no striking clinical differences in other variables between the ARB and the ACEI groups.

### 3.2. Comparison of Hyperkalemia Occurrence and Onset Time

**Figure 2:**
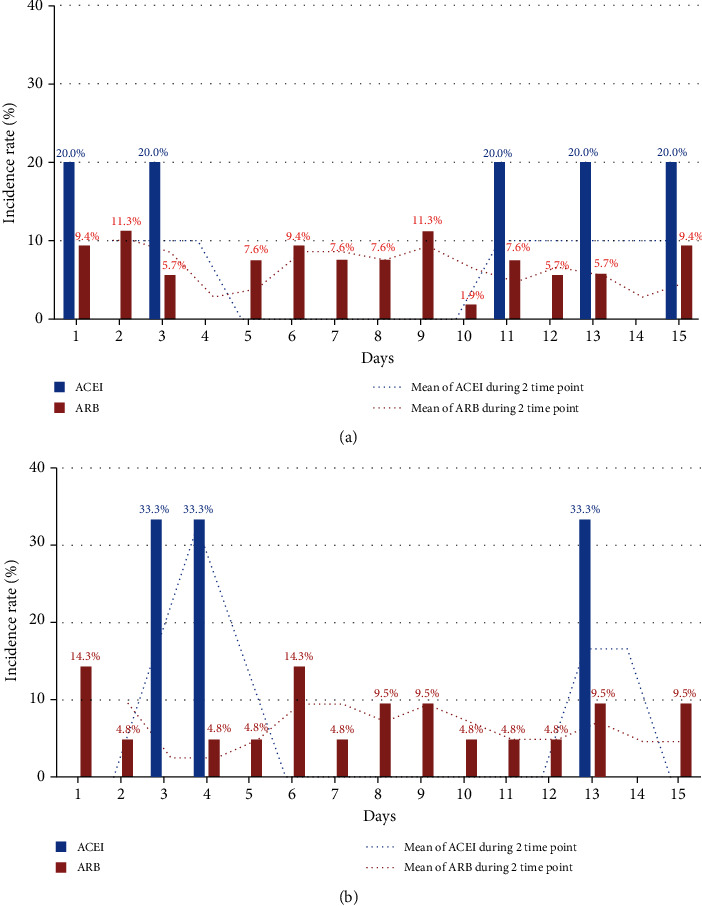
Occurrence of hyperkalemia during hospitalization after ACEI or ARB use at (a) >5.5 mEq/L and (b) 6.0 mEq/L cutoff.

Serum potassium levels for monitoring hyperkalemia after initiation of ACEI and ARB were recorded daily for 15 days in hospitalized patients. The Kaplan-Meier survival curve shows the comparison of hyperkalemia prevalence between the ACEI and ARB groups, and there was no significant difference in the survival probability between the two groups because the log-rank test *p* value was 0.253 at the 5.5 mEq/L standard. Hyperkalemia was detected in 0.9% of the patients (5/562) in the ACEI group and occurred on days 1, 3, 11, 13, and 15 at the 5.5 mEq/L cutoff of hyperkalemia, while 2.1% of the patients (53/2539) were observed to have hyperkalemia in the ARB group, the highest percentage being 11.3% (6 patients) on days 2 and 9, and 9.4% (5 patients) on days 1, 6, and 15 (Figure 2(a)). However, since the number of cases of hyperkalemia was very small, there was no significant difference between different days of onset. The log-rank test *p* value was 0.054 at the 5.5 mEq/L and 0.877 at the 6.0 mEq/L standard, indicating a lack of statistical significance. Hyperkalemia was detected in 0.5% of the patients (3/562) in the ACEI group, on days 3, 4, and 13, at the 6.0 mEq/L cutoff of hyperkalemia (Figure 2(b)). In the ARB group, hyperkalemia occurred in 0.8% of the patients (21/2539), and the highest level was found in 3 patients (14.3%) on days 1 and 6; the rest showed evenly distributed occurrence throughout.

### 3.3. Relationship between Hyperkalemia and Antihypertensive Drug Use

The highest incidence of hyperkalemia according to the ARB type was associated with the use of eprosartan 4.7% (4/85 cases), valsartan 3.9% (16/408 cases), and olmesartan 3.2% (7/216 cases) at the 5.5 mEq/L potassium standard. Incidences of hyperkalemia with the use of valsartan were the highest at 1.5% (6/408 persons), followed by olmesartan (1.4%, 3/216) and irbesartan (0.9%, 2/222) at the 6.0 mEq/L potassium. However, the overall incidence of hyperkalemia was very low, and there was no statistically significant difference according to the type of ARB.

Several factors affecting the development of hyperkalemia were investigated for 15 days after ACEI and ARB administration (Table 2). When the cutoff of hyperkalemia was 5.5 mEq/L, there was no significant difference in the incidence of hyperkalemia between ARB and ACEI groups (HR = 1.33 (95%CI = 0.53–3.35), *p* = 0.547), and this was also true in cases where the cutoff was 6.0 mEq/L (HR = 0.84 (95%CI = 0.25–2.84), *p* = 0.774). The incidence of hyperkalemia increased significantly (7.3 times) as the reference level (1.2 mEq) of creatinine increased (HR = 7.31, 95%CI = 4.19–12.76, *p* < 0.001) at the 5.5 mEq/L serum potassium level and 35.1 times (HR = 35.11, 95%CI = 8.25–149.52, *p* < 0.001) at the 6.0 mEq/L level. The incidence of hyperkalemia was higher with lower MDRD-eGFR values, and the incidence at 60 mL/min/1.73 m^2^ was 13 times higher (HR = 13.06, 95%CI = 4.05–42.10, *p* < 0.001) than that at 90 mL/min/1.73 m^2^ at the 5.5 mEq/L potassium level. BUN, MDRD-eGFR, and creatinine variables were omitted because of multicollinearity. The incidence of hyperkalemia was 5.7 times higher (HR = 5.72, 95%CI = 3.24–10.12, *p* < 0.001) at the 5.5 mEq/L potassium cutoff and 9.2 times higher (HR = 9.16, 95%CI = 4.02–20.88, *p* < 0.001) at the 6.0 mEq/L potassium cutoff when the patients had chronic renal failure. There was no significant correlation between other comorbidities and hyperkalemia incidence. Moreover, for drugs such as statins and CCB, which are commonly administered with ACEI (or ARB), there was no effect on hyperkalemia.

## 4. Discussion

This study is primarily aimed at retrospectively analyzing when and how much ACEI- or ARB-induced hyperkalemia occurred after initiating use based on EMR data. In this study, the incidence of ACEI-induced hyperkalemia was 0.5–0.9%, and the incidence of ARB-induced hyperkalemia was 0.8–2.1%. The usual concerns associated with the study population in a cohort study are not applicable here, as there is no significant difference in incidence for ACEI- (or ARB-) induced hyperkalemia compared to that reported in previous studies [5, 10].

Because the levels of serum potassium—defined as hyperkalemia—in the study varied, varying results were inevitable [12]. For this reason, we set the cutoff of hyperkalemia to two levels, 5.5 mEq/L and 6.0 mEq/L. In a related previous study, in 52.4% of the patients, hyperkalemia occurred within 1 week of the initiation of the drug during a 30-day observation period, and in 62.4% of the patients, hyperkalemia occurred within 1 week of the initiation of the drug during a 15-day observation period. In particular, the study found that hyperkalemia occurred with the highest rate on the first day (10.2% for the 30-day observation period and 12.1% for the 15-day observation period) [13]. However, in our study, hyperkalemia occurred uniformly throughout the 15-day observation period, and occurrence within the first week during the 15 days was 50.9% at the cutoff of 5.5 mEq/L and 47.6% at the cutoff of 6.0 mEq/L potassium level. This was a large difference as compared with 62.4% in the previous study. In addition, the fraction occurring on the first day was 9.4%, which was less than the 10% at the cutoff of 5.5 mEq/L during the 15-day observation period, which in turn was lower than that observed in the previous study (12.1%) [14] and the same as the fraction occurring on the last day of observation (day 15). This is different from the results of the previous studies that monitored the serum potassium level immediately after the start of drug use [10] and seemed to be significant in that it can suggest the need for large-scale prospective observational studies in the future. In other words, according to the present study, hyperkalemia may occur at any time within 15 days of the first administration, and hence, more research is needed to identify monitoring schedules for hyperkalemia in the early stage.

Factors affecting hyperkalemia incidence include diabetes mellitus, tissue injury, inadequate diet, medication, and deficiency of hormones related to the above conditions [15]. Hyperkalemia occurs most readily when there is renal dysfunction because kidneys play an important role in potassium homeostasis, where 90% of the excess potassium is excreted specifically through the kidneys [16]. The results of this study adhere to the above theory, since, according to our observations, the factors associated with abnormal renal function [17], among the various possible factors, showed the most significant impact on the development of ACEI- or ARB-induced hyperkalemia [18]. That is, the incidence of hyperkalemia was higher in the presence of higher creatinine, lower MDRD-eGFR, and comorbid renal failure. However, this could have lead to exaggerated results because the number of events is small, and the multivariate comparison did not yield significant results in the case of creatinine and MDRD-eGFR. In MDRD-eGFR, the number of hyperkalemia incidences was too small for an individual grade-level analysis. Specifically, there were too few cases for the cutoff of 6.0 mEq/L, so a statistical analysis could not be performed. By additionally stratifying the analysis by baseline potassium level, we confirmed that there were no significant between-group differences.

In most previous studies, observations have been scant on the specific onset time of ARB-induced hyperkalemia [19]. Therefore, there is a need to be cautious in interpreting the results presented in the previous study showing that the highest incidence of hyperkalemia occurs within 15 days, since they were inconsistent with ours. Most previous studies on ARB-induced hyperkalemia have focused on methods of measuring potassium levels after administration of ACEI or ARB. The number of times that potassium levels were measured during a given period of one year or three years [20] and the relationship between that number and the incidence of hyperkalemia, the factors for potassium monitoring (age, number of outpatient visits, hospitalization, sex, race, and baseline potassium level of subjects) [21], and the comorbidities that require meticulous monitoring of potassium levels [22] were investigated. Moreover, a score-based study has been performed to help predict hyperkalemia occurrence using baseline characteristics [23]. This was conducted for more meaningful measurement of the potassium level considering that the incidence of hyperkalemia is not that high because excessively close monitoring of the potassium level may lead to overtreatment and wastage of medical expenses as well as increased discomfort for the patients.

The limitations of this study lie in that it is an EMR-based real-world evidence (RWE) study rather than a randomized controlled trial (RCT). ARB- (or ACEI-) induced hyperkalemia may occur at any point during drug use, but this study was conducted within a limited timeframe of 15 days. Therefore, we did not include cases of hyperkalemia that occurred beyond this range. The bimodal action of ACE inhibitors should be considered in advance. Despite this, our results are sufficient to refute the previous study that showed that most hyperkalemia cases occur within one week after initiation. Therefore, the 15-day period can be enough considering the special conditions that need to be monitored daily. The second limitation is that, since the number of cases of hyperkalemia is quite low, meaningful statistical evaluation is not possible. There is no trend in the onset of action of hyperkalemia within the first 15 days after administration. Since the incidence rate of hyperkalemia > 6 mEq/L was less than 1% of the total, it would be difficult to analyze more precisely. However, one of the great advantages of RWE is that it is easy to access the rare side effects. In fact, if the incidence rate is low (1–2%) after admission as in this study, access through RCT would have been limited. Although this does not provide a statistical meaning for various conditions, previous studies have been complemented by RWE data. Lastly, other studies have considered the intake of potassium from diet, supplements, or drugs as a possible cause of hyperkalemia [24], and this study fails to take these factors into consideration.

Since EMR uses previously accumulated data, this study is extremely advantageous as it provides an analysis of rare diseases or rare adverse drug reactions. In particular, this study was able to approach the incidence of adverse effects with minimal bias as hyperkalemia is clearly represented by structured numbers compared to the EMR-based retrospective cohort study with more bias that could produce different results for each study. In particular, thinking about the timing of potassium level measurement for proper monitoring from excessive potassium monitoring for hyperkalemia is one of the important significances of this study. Another significance is to reduce errors that overemphasize only the early occurrence of hyperkalemia. However, when prescribing ACEI or ARB in patients with abnormal renal function, clinicians should be cognizant of the possibility of hyperkalemia development, and periodic follow-ups are warranted. However, further large-scale studies are needed in the future, since the incidence of hyperkalemia is low, and a trend for onset time after ACEI or ARB use remains unclear.

## Figures and Tables

**Table 1:** Baseline characteristics of the patients (*n* = 3101).

	ACEI	ARB	*p* value
Number, *n* (%)	562 (18.1%)	2539 (81.9%)	
Age, years	62 ± 12	63 ± 12	0.068
Female, *n* (%)			
BMI, kg/m^2^	23.8 ± 3.3	24.5 ± 3.6	0.001
BUN, mg/dL	17.1 ± 8.9	19.1 ± 12.4	<.001
Creatinine, mg/dL	1.0 ± 1.2	1.3 ± 1.7	<.001
MDRD-eGFR, mL/min/1.73 m^2^	82.1 ± 24.1	76.3 ± 26.9	<.001
MDRD-eGFR, *n* (%)			<.001
≥90 mL/min/1.73 m^2^	189 (33.6)	718 (28.3)	
60-89 mL/min/1.73 m^2^	297 (52.9)	1297 (51.1)	
<60 mL/min/1.73 m^2^	76 (13.5)	524 (20.6)	
Sodium, mEq/L	140 ± 3	140 ± 4.0	0.711
Potassium, mEq/L	4.2 ± 0.4	4.3 ± 0.5	0.009
Comorbidity, *n* (%)			
Heart failure	5 (0.9)	35 (1.4)	0.353
Hypertensive disease	29 (5.2)	133 (5.2)	0.940
Ischemic heart disease	303 (53.9)	978 (38.5)	<.001
Cerebrovascular disease	27 (4.8)	163 (6.4)	0.148
Diabetes mellitus	36 (6.4)	133 (5.2)	0.270
Acute renal failure	7 (1.3)	109 (4.3)	0.001
Cancer	43 (7.7)	274 (10.8)	0.026
Medication, *n* (%)			
*β*-Blocker	217 (38.6)	782 (30.8)	<.001
CCB	161 (28.7)	1157 (45.6)	<.001
Statin	437 (77.8)	1565 (61.6)	<.001

ACEI, angiotensin converting enzyme inhibitor; ARB, angiotensin receptor blocker; BMI, body mass index; BUN, blood urea nitrogen; CCB, calcium channel blocker; GFR, glomerular filtration rate, MDRD, modification of diet in renal disease.

**Table 2:** Causal relationship between hyperkalemia and antihypertensive drug use.

	Serum potassium > 5.5 mEq/L				Serum potassium > 6.0 mEq/L			
	Univariate		Multivariate		Univariate		Multivariate	
	HR (95% CI)	*p* value	HR (95% CI)	*p* value	HR (95% CI)	*p* value	HR (95% CI)	*p* value
Antihypertensive drug								
ACE inhibitor	1.0 (reference)		1.0 (reference)		1.0 (reference)		1.0 (reference)	
ARB	1.69 (0.68-4.24)	0.261	1.33 (0.53-3.35)	0.547	1.10 (0.33-3.69)	0.878	0.84 (0.25-2.84)	0.774
Age	0.99 (0.97-1.01)	0.373			0.98 (0.96-1.01)	0.246		
Age (≥60 years)	0.76 (0.45-1.30)	0.314			0.66 (0.29-1.50)	0.322		
Female	0.58 (0.32-1.05)	0.073			0.36 (0.12-1.06)	0.065		
BUN	1.03 (1.03-1.04)	<.001			1.03 (1.02-1.04)	<.001		
Creatinine	1.23 (1.17-1.29)	<.001			1.22 (1.14-1.31)	<.001		
Creatinine (>1.2 mg/dL)	7.31 (4.19-12.76)	<.001			35.11 (8.25-149.52)	<.001		
MDRD	0.96 (0.95-0.97)	<.001			0.95 (0.93-0.97)	<.001		
MDRD		<.001						
≥90 mL/min/1.73 m^2^	1.0 (reference)				1.0 (reference)			
60-89 mL/min/1.73 m^2^	2.23 (0.63-7.90)	0.214			N/A			
<60 mL/min/1.73 m^2^	13.06 (4.05-42.10)	<.001			N/A			
Sodium	0.91 (0.88-0.95)	<.001			0.92 (0.87-0.98)	0.014		
Comorbidity								
Heart failure	0.83 (0.12-6.02)	0.856			N/A			
Hypertensive disease	1.77 (0.64-4.89)	0.270			1.03 (0.14-7.63)	0.977		
Ischemic heart disease	0.25 (0.09-0.68)	0.007			0.31 (0.07-1.31)	0.112		
Cerebrovascular disease	0.82 (0.26-2.63)	0.742			1.40 (0.33-5.93)	0.652		
Diabetes mellitus	0.71 (0.17-2.93)	0.640			0.89 (0.12-6.63)	0.913		
Acute renal failure	6.51 (3.76-11.29)	<.001	5.72 (3.24-10.12)	<.001	9.01 (3.99-20.37)	<.001	9.16 (4.02-20.88)	<.001
Cancer	0.45 (0.18-1.14)	0.091			0.99 (0.34-2.89)	0.979		
Medication								
*β*-Blocker	0.60 (0.30-1.19)	0.143			0.42 (0.12-1.40)	0.155		
CCB	1.48 (0.87-2.50)	0.149			2.36 (0.98-5.71)	0.056		
Statin	0.50 (0.29-0.86)	0.013	0.68 (0.38-1.20)	0.177	0.42 (0.18-1.03)	0.057		

BUN, MDRD, and creatinine variables are omitted because of multicollinearity.

## Data Availability

The data that support the findings of this study are available from the corresponding author upon reasonable request.

## References

[1] Grassi G., Calhoun D. A., Mancia G., Carey R. M. (2019). Resistant hypertension management: Comparison of the 2017 American and 2018 European High Blood Pressure Guidelines. *Current Hypertension Reports*.

[2] Wang K., Hu J., Luo T. (2018). Effects of angiotensin-converting enzyme inhibitors and angiotensin II receptor blockers on all-cause mortality and renal outcomes in patients with diabetes and albuminuria: a systematic review and meta-analysis. *Kidney and Blood Pressure Research*.

[3] Xie X., Liu Y., Perkovic V. (2016). Renin-angiotensin system inhibitors and kidney and cardiovascular outcomes in patients with CKD: a Bayesian network meta-analysis of randomized clinical trials. *American Journal of Kidney Diseases*.

[4] Krittanawong C., Kitai T. (2017). Pharmacogenomics of angiotensin receptor/neprilysin inhibitor and its long-term side effects. *Cardiovascular Therapeutics*.

[5] Desai A. (2008). Hyperkalemia associated with inhibitors of the renin-angiotensin-aldosterone system: balancing risk and benefit. *Circulation*.

[6] Svensson M., Gustafsson F., Galatius S., Hildebrandt P. R., Atar D. (2004). How prevalent is hyperkalemia and renal dysfunction during treatment with spironolactone in patients with congestive heart failure?. *Journal of Cardiac Failure*.

[7] Pham A. Q., Sexton J., Wimer D., Rana I., Nguyen T. (2017). Managing hyperkalemia: stepping into a new frontier. *Journal of Pharmacy Practice*.

[8] Epstein M. (2016). Hyperkalemia: current concepts and emerging therapeutic options. *Kidney International Supplements*.

[9] Weir M. R., Rolfe M. (2010). Potassium homeostasis and renin-angiotensin-aldosterone system inhibitors. *Clinical Journal of the American Society of Nephrology*.

[10] Park I.-W., Sheen S. S., Yoon D. (2014). Onset time of hyperkalaemia after angiotensin receptor blocker initiation: when should we start serum potassium monitoring?. *Journal of Clinical Pharmacy and Therapeutics*.

[11] Levey A. S., Bosch J. P., Lewis J. B., Greene T., Rogers N., Roth D. (1999). A more accurate method to estimate glomerular filtration rate from serum creatinine: a new prediction equation. *Annals of Internal Medicine*.

[12] Ficheur G., Chazard E., Beuscart J.-B., Merlin B., Luyckx M., Beuscart R. (2014). Adverse drug events with hyperkalaemia during inpatient stays: evaluation of an automated method for retrospective detection in hospital databases. *BMC Medical Informatics and Decision Making*.

[13] Bandak G., Sang Y., Gasparini A. (2017). Hyperkalemia after initiating renin–angiotensin system blockade: the Stockholm creatinine measurements (SCREAM) Project. *Journal of the American Heart Association*.

[14] Chang A. R., Sang Y., Leddy J. (2016). Antihypertensive medications and the prevalence of hyperkalemia in a large health system. *Hypertension*.

[15] Nilsson E., Gasparini A., Ärnlöv J. (2017). Incidence and determinants of hyperkalemia and hypokalemia in a large healthcare system. *International Journal of Cardiology*.

[16] Arnold R., Pianta T. J., Pussell B. A., Endre Z., Kiernan M. C., Krishnan A. V. (2019). Potassium control in chronic kidney disease: implications for neuromuscular function. *Internal Medicine Journal*.

[17] Kang H., Hong S. H. (2019). Risk of kidney dysfunction from polypharmacy among older patients: a nested case-control study of the South Korean senior cohort. *Scientific Reports*.

[18] Han S.-W., Won Y.-W., Yi J.-H., Kim H.-J. (2007). No impact of hyperkalaemia with renin-angiotensin system blockades in maintenance haemodialysis patients. *Nephrology Dialysis Transplantation*.

[19] Raebel M. A. (2012). Hyperkalemia associated with use of angiotensin-converting enzyme inhibitors and angiotensin receptor blockers. *Cardiovascular Therapeutics*.

[20] Abbas S., Ihle P., Harder S., Schubert I. (2015). Risk of hyperkalemia and combined use of spironolactone and long-term ACE inhibitor/angiotensin receptor blocker therapy in heart failure using real-life data: a population- and insurance-based cohort. *Pharmacoepidemiology and Drug Safety*.

[21] Malta D., Arcand J., Ravindran A., Floras V., Allard J. P., Newton G. E. (2016). Adequate intake of potassium does not cause hyperkalemia in hypertensive individuals taking medications that antagonize the renin angiotensin aldosterone system. *The American Journal of Clinical Nutrition*.

[22] Raebel M. A., McClure D. L., Simon S. R. (2007). Laboratory monitoring of potassium and creatinine in ambulatory patients receiving angiotensin converting enzyme inhibitors and angiotensin receptor blockers. *Pharmacoepidemiology and Drug Safety*.

[23] Maciejewski M. L., Hammill B. G., Qualls L. G., Hastings S. N., Wang V., Curtis L. H. (2016). Appropriate baseline laboratory testing following ACEI or ARB initiation by Medicare FFS beneficiaries. *Pharmacoepidemiology and Drug Safety*.

[24] Kovesdy C. P. (2015). Management of hyperkalemia: an update for the internist. *The American Journal of Medicine*.

